# Limited discriminative performance of endoscopic deep learning for *Helicobacter pylori* status assessment in gastric cancer patients: a retrospective study

**DOI:** 10.3389/fgstr.2026.1878650

**Published:** 2026-07-15

**Authors:** Wenyi Zhou, Yixing Wang, Yuhong Wang, Yongluo Jiang, Wencan He, Binbin Xu

**Affiliations:** 1 Radiology Department, Changsha Stomatological Hospital, Changsha, China; 2 Department of Medical Oncology, Sun Yat-sen University Cancer Center; State Key Laboratory of Oncology in South China, Guangdong Provincial Clinical Research Center for Cancer, Guangzhou, China; 3 Department of Endoscopy, Sun Yat-sen University Cancer Center; State Key Laboratory of Oncology in South China, Guangdong Provincial Clinical Research Center for Cancer, Guangzhou, China; 4 Department of Nuclear Medicine, Sun Yat-sen University Cancer Center; State Key Laboratory of Oncology in South China, Guangdong Provincial Clinical Research Center for Cancer, Guangzhou, China; 5 Precision Medicine Institute, The First Affiliated Hospital, Sun Yat-sen University, Guangzhou, China; 6 EuroMov Digital Health in Motion, Univ Montpellier, IMT Mines Ales, Ales, France

**Keywords:** endoscopic imaging, gastric cancer, *Helicobacter pylori*, image classification, neural networks

## Abstract

**Objectives:**

*Helicobacter pylori* infection is a major risk factor for gastric cancer, but evidence for predicting *H. pylori* status from endoscopic images in patients with established gastric cancer remains limited. We evaluated the performance of endoscopic image-based classification models for *H. pylori* status in a retrospective gastric cancer cohort.

**Methods:**

We retrospectively collected 602 endoscopic images from 337 patients with gastric cancer treated at a tertiary cancer center. After preprocessing and quality control, 576 images from 329 patients were retained for model development and evaluation. *Helicobacter pylori* status was defined using routine clinical testing, including serum anti-*H. pylori* IgG and/or the ^13^C urea breath test. Endoscopic image classification models were evaluated across repeated stratified train, validation, and test splits. The primary performance metric was the area under the receiver operating characteristic curve (AUC).

**Results:**

Model discrimination was limited. The best-performing approach achieved a median test AUC of 0.6255, with sensitivity of 0.7308, specificity of 0.3714, accuracy of 0.6092, and F1 score of 0.6800. Fine-tuning showed a higher median AUC than retraining, but discrimination remained limited in both strategies.

**Conclusions:**

In this retrospective gastric cancer cohort, endoscopic image-based classification showed limited ability to discriminate *H. pylori* status. These findings suggest that image-based assessment of *H. pylori* status in patients with gastric cancer remains challenging and may require more rigorous phenotyping, larger datasets, and validation across clinically diverse populations.

## 1 Introduction

*Helicobacter pylori* (*H. pylori*) is a human-specific pathogen and a major risk factor for several gastric conditions, including peptic ulcers, gastritis, mucosa-associated lymphoid tissue lymphoma, and gastric cancer (1, 2). Recognized as a carcinogen, *H. pylori* plays a central role in gastric carcinogenesis, typically through chronic inflammation of the gastric mucosa that may progress over time to atrophy, dysplasia, and eventually cancer (3). Early detection and treatment of *H. pylori*, therefore, remain important components of gastric cancer prevention strategies (3, 4).

In clinical practice, common methods for detecting *H. pylori* include gastric biopsies obtained during endoscopy followed by histology, culture, polymerase chain reaction, or rapid urease testing, as well as non-invasive approaches such as serology and the urea breath test (2, 4). Although these methods are clinically useful, they may require additional procedures, costs, or time.

Recently, deep neural networks have been widely applied to complex clinical image classification tasks, including radiologic image interpretation (5) and endoscopic image analysis (6–10). Contemporary medical image analysis has also increasingly explored transformer-based and multimodal approaches, especially for tasks requiring long-range visual context or integration of complementary clinical information (11, 12). In upper gastrointestinal endoscopy, deep learning has been investigated for image-based detection of *H. pylori* infection (6, 7). Previous studies have reported encouraging results. For example, Shichijo et al. (13) described a convolutional neural network with an area under the receiver operating characteristic curve (AUC) of approximately 0.89, and Itoh et al. (14) also reported high performance in a separate cohort. More generally, systematic review data suggest that artificial intelligence may have potential for endoscopic prediction of *H. pylori* status (7).

At the same time, evidence in gastric cancer patients remains limited. This population is clinically important because cancer-related mucosal changes, surface irregularity, bleeding, severe atrophy, intestinal metaplasia, and post-eradication findings may coexist and may affect endoscopic appearance (15). In addition, the Kyoto classification literature indicates that endoscopic findings may reflect not only current infection but also past infection and long-standing mucosal remodeling (16). These considerations suggest that assessment of *H. pylori* status from endoscopic images may be more challenging in gastric cancer patients and warrants further study.

The aim of the present study was to evaluate the performance of deep learning-based classification of endoscopic images for *H. pylori* status in gastric cancer patients. Using a retrospective cohort with patient-level clinical testing for *H. pylori* status and a limited number of images per patient, we examined the discriminative ability of image-only classifiers in this specific clinical setting.

## 2 Methods

### 2.1 Patients, images, and reference standard

This retrospective study used archived upper gastrointestinal endoscopy still images from gastric cancer patients. The initial dataset contained 602 images from 337 patients, with a median image dimension of 536 × 708 pixels. Of these patients, 201 were classified as *H. pylori* positive and 136 as *H. pylori* negative. The initial image set included 361 images in the positive group and 241 images in the negative group. Mean age was 56.6 years in the positive group and 58.3 years in the negative group.

The images were exported from routine clinical endoscopy records after removal of embedded patient-identifying metadata for privacy protection. Because this was a retrospective chart-based study using anonymized exported images, device-level acquisition parameters, illumination settings, and processor-specific image enhancement settings could not be systematically reconstructed. No prospective image acquisition protocol was imposed. Therefore, the dataset reflects the heterogeneity of routine clinical endoscopic imaging rather than standardized research image acquisition.

All patients had been evaluated for *H. pylori* by at least one routine clinical test recorded in the medical chart: serum anti-*H. pylori* IgG and/or the ^13^C urea breath test. Patients were classified as *H. pylori* positive when either test was positive. This pragmatic chart-based definition reflects routine clinical practice in a retrospective cohort. We note, however, that the two tests do not represent identical biological information: serology may remain positive after previous exposure or eradication, whereas the urea breath test more directly reflects current urease activity (4, 15).

### 2.2 Image preprocessing and quality control

A major preprocessing step was image segmentation. Although upper gastrointestinal endoscopy follows common procedural standards, stored clinical images may vary substantially in quality and composition. Technical limitations of endoscopy equipment, lens distortion, variable aspect ratios, mucus, foreign bodies, instruments, and embedded procedure-related text may all affect image quality and create spurious features for neural network models.

To reduce these non-mucosal signals, regions of interest were first identified using the largest Segment Anything Model (17), which has demonstrated high performance on similarly challenging medical images, such as those of trachoma-infected eyes (18). The segmentation step was used to retain analyzable mucosal regions and suppress non-mucosal image components, including borders and residual procedure-related text such as dates or identifiers. Images with lens distortion or distorted aspect ratio were corrected using image processing techniques based on region circularity. Images that were corrupted, severely distorted, or dominated by foreign bodies, instruments, residual text, or non-mucosal content were excluded.

**Figure 1:**
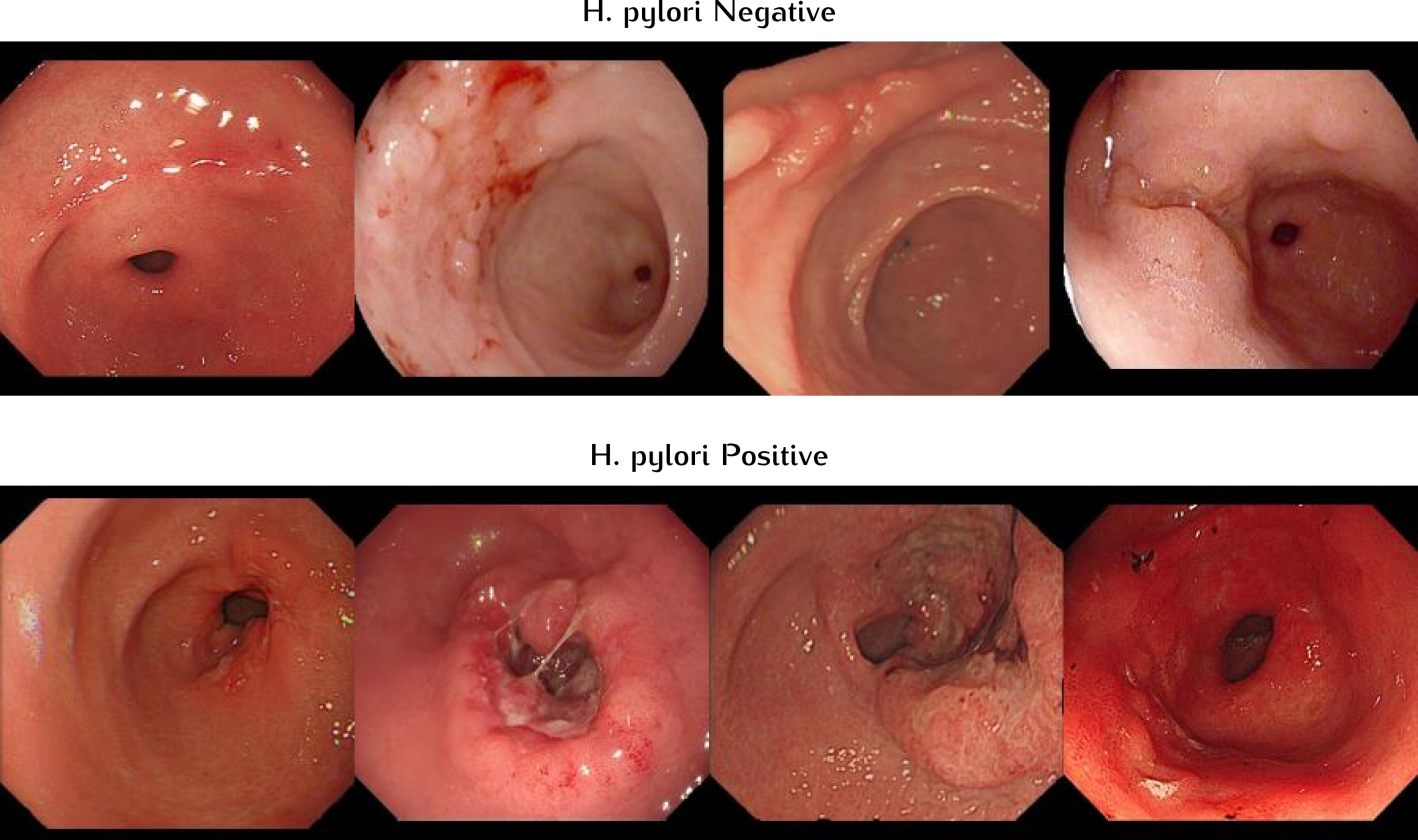
Representative *H. pylori*-negative and *H. pylori*-positive endoscopic images.

After data cleaning and preprocessing, 576 images from 329 patients remained for model development. The final image set was manually inspected before training. Sample images are shown in **Figure 1**.

### 2.3 Model development

Five EfficientNet backbones, from B0 to B4, were evaluated for binary classification (19). EfficientNet was selected as a contemporary, computationally efficient image-classification family rather than as a claim of architectural novelty. The aim was therefore not to benchmark competing computer vision architectures, but to evaluate whether a parameter-efficient convolutional model family could extract a meaningful image-only signal from this gastric cancer cohort. All models used ImageNet-pretrained weights and the same classification head, consisting of global average pooling, a dense layer with 128 units and ReLU activation, a dense layer with 32 units and ReLU activation, and a final sigmoid output node.

Two transfer-learning strategies were compared. In the retraining strategy, the network backbone was updated from pretrained initialization. In the fine-tuning strategy, the pretrained backbone was kept fixed, and only the newly added classification head was optimized. This comparison was retained because it is methodologically informative in modest-sized medical datasets: full backbone updating tests whether the cohort-specific image signal is learnable when all trainable layers are optimized, whereas the fixed-backbone fine-tuning strategy constrains the number of trainable parameters and may reduce overfitting to a limited cohort (20).

### 2.4 Training and evaluation

Images were decoded as three-channel JPEG images, resized to the input resolution required by each EfficientNet backbone, and processed using the standard EfficientNet preprocessing function. Data augmentation was applied during training and included random horizontal and vertical flipping and random rotation. Models were trained using TensorFlow/Keras with stochastic gradient descent, a learning rate of 0.001, a momentum of 0.9, and a binary cross-entropy loss. The batch size was 16, and training was run for 500 epochs. Dropout and early stopping were not used because the objective was to compare the full training trajectories of the two strategies across repeated runs rather than to select a single early-stopped checkpoint. Training was performed on an NVIDIA RTX A4000 GPU.

To reduce dependence on a single random partition, performance was summarized over 10 repeated train, validation, and test runs with different random seeds, using a stratified 70%/15%/15% split in each run. Splitting was performed at the patient level, so that eventual multiple images from a given patient were assigned to the same partition. This approach was used to reduce the risk of data leakage between training and test sets, in line with published guidance for machine learning studies in gastrointestinal endoscopy (20). The primary endpoint was the median test area under the receiver operating characteristic curve (AUC). Accuracy, sensitivity, specificity, and F1 score were also recorded. Test-set loss, accuracy, AUC, sensitivity, specificity, and F1 score were logged at each epoch. For each metric, 95% bootstrap confidence intervals were estimated across the 10 repeated runs as an exploratory summary of between-run variability due to random partitioning and model initialization.

## 3 Results

After preprocessing and quality control, 576 endoscopic images from 329 patients remained for model development and evaluation.

Performance was assessed over 10 repeated runs and summarized using median values on the test set. The primary metric was the area under the receiver operating characteristic curve (AUC), with accuracy, sensitivity, and specificity also recorded.

Across all EfficientNet variants, fine-tuning showed higher median AUC than retraining. In the retraining setting, median test AUC values ranged from 0.5791 to 0.6025, with the best performance observed for EfficientNetB3. In the fine-tuning setting, median test AUC values ranged from 0.6025 to 0.6255, with the best performance observed for EfficientNetB0. Thus, although fine-tuned models showed higher median AUC than their retrained counterparts, none achieved strong discrimination.

**Figure 2:**
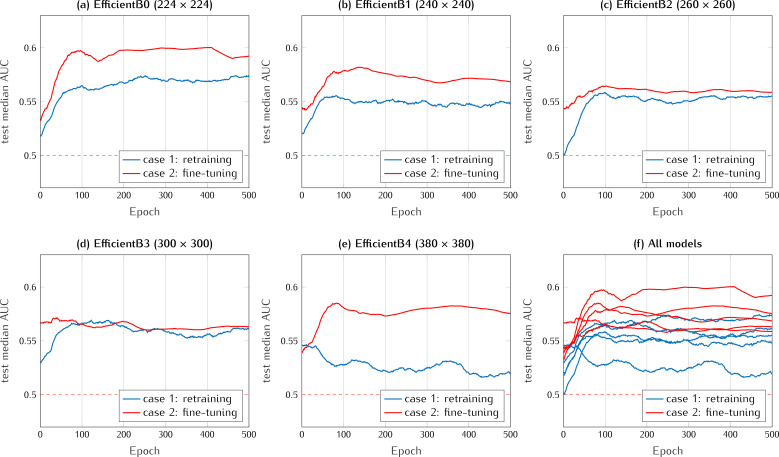
Comparison of test-set AUC between the two training strategies, retraining and fine-tuning, across individual EfficientNet models **(A–E)** and the pooled analysis **(F)**. Curves show median test AUC across 10 repeated stratified train, validation, and test runs with different random seeds. The dashed horizontal line indicates chance-level performance (AUC = 0.50).

**Figure 2** shows the AUC trends for retraining and fine-tuning across the five EfficientNet backbones and in the pooled analysis. Fine-tuning consistently yielded higher AUC values over training epochs and showed a smoother trajectory than retraining. This pattern was also observed for EfficientNetB4, which performed relatively poorly in the retraining setting but showed improved and more stable behavior with fine-tuning.

The median performance measures are summarized in **Table 1**. The best overall configuration by AUC was fine-tuned EfficientNetB0, which achieved an accuracy of 0.6092, an AUC of 0.6255, a specificity of 0.3714, a sensitivity of 0.7308, and an F1 score of 0.6800. Fine-tuned EfficientNetB1 showed a higher specificity of 0.5143, but this was accompanied by a lower AUC of 0.6107.

**Table 1:** Repeated-run test-set performance of the evaluated EfficientNet models under retraining and fine-tuning.

	Model	ACC	AUC	Specificity	Sensitivity	F1 score
Retraining	EfficientNetB0	0.5977	0.6004	0.3857	0.7308	0.6877
		[0.592, 0.638]	[0.530, 0.632]	[0.371, 0.414]	[0.692, 0.779]	[0.652, 0.711]
	EfficientNetB1	0.6034	0.5926	0.3429	0.7308	0.6820
		[0.540, 0.638]	[0.498, 0.616]	[0.271, 0.443]	[0.712, 0.788]	[0.649, 0.718]
	EfficientNetB2	0.5747	0.5857	0.3286	0.7115	0.6609
		[0.540, 0.603]	[0.495, 0.600]	[0.286, 0.386]	[0.692, 0.750]	[0.643, 0.678]
	EfficientNetB3	0.5977	0.6025	0.3571	0.7596	0.6902
		[0.575, 0.632]	[0.526, 0.637]	[0.314, 0.414]	[0.721, 0.788]	[0.657, 0.716]
	EfficientNetB4	0.5920	0.5791	0.1857	0.8654	0.7286
		[0.575, 0.621]	[0.502, 0.608]	[0.071, 0.257]	[0.837, 0.942]	[0.708, 0.737]
Fine-tuning	EfficientNetB0	0.6092	0.6255	0.3714	0.7308	0.6800
		[0.575, 0.632]	[0.542, 0.633]	[0.343, 0.600]	[0.654, 0.846]	[0.638, 0.713]
	EfficientNetB1	0.5920	0.6107	0.5143	0.7019	0.6707
		[0.529, 0.644]	[0.549, 0.636]	[0.257, 0.686]	[0.423, 0.875]	[0.518, 0.735]
	EfficientNetB2	0.5690	0.6025	0.3286	0.6923	0.6629
		[0.552, 0.586]	[0.508, 0.638]	[0.243, 0.514]	[0.625, 0.808]	[0.623, 0.689]
	EfficientNetB3	0.5690	0.6037	0.4571	0.6250	0.6185
		[0.517, 0.609]	[0.557, 0.635]	[0.357, 0.571]	[0.490, 0.750]	[0.553, 0.691]
	EfficientNetB4	0.5805	0.6074	0.4857	0.5962	0.6228
		[0.552, 0.609]	[0.555, 0.633]	[0.343, 0.629]	[0.519, 0.827]	[0.593, 0.723]

These results indicate limited class separation and suggest that the improvement associated with fine-tuning was not sufficient to yield clinically useful discrimination.

## 4 Discussion

This study evaluated whether contemporary transfer-learning classifiers retain discriminative value for *H. pylori* status in a clinically complex gastric cancer cohort. The best observed discrimination, with a median AUC of 0.6255, indicated limited ability to separate labeled positive from labeled negative images in this setting. Although performance was above the no-skill level, it remained substantially below what would usually be considered adequate for clinical decision support.

An important implication is that previously reported high performance for endoscopic *H. pylori* classification should not be generalized uncritically to gastric cancer populations (7, 13, 14). Differences in cohort composition, image acquisition, and underlying gastric pathology may influence model performance, and in the present gastric cancer cohort, classification remained challenging.

The limited performance observed here should not be attributed to a single cause. Gastric cancer patients plausibly represent a domain shift relative to many previously studied *H. pylori* datasets. Endoscopic correlates of infection can overlap with chronic mucosal injury, severe atrophy, intestinal metaplasia, tumor-associated change, and post-eradication patterns (3, 15, 16). The Kyoto classification literature distinguishes current infection from past infection and indicates that some endoscopic features may persist after infection status has changed (15, 16). Consequently, the association between image appearance and a binary infection label may be weak or noisy in this subgroup.

The reference standard also likely contributed to this difficulty. In routine practice, combining serology and the urea breath test is pragmatic, but it may mix active infection with prior exposure (4). A model trained against such a combined endpoint may therefore be asked to predict a label that does not correspond to one stable endoscopic phenotype. This issue should not be viewed only as a technical weakness. It is also a clinical reality of retrospective routine-care datasets. For that reason, the present limited-performance result remains informative.

For each model, the first row reports the median performance across 10 repeated stratified runs, and the second row reports the 95% bootstrap confidence interval across runs.

The comparison between retraining and fine-tuning was secondary but still useful. Fine-tuning showed consistently higher median AUC than retraining, which is consistent with the expectation that smaller medical imaging datasets may benefit more from restrained transfer learning than from full backbone updating (20). However, the absolute performance remained limited in both settings. The main message is therefore not that one EfficientNet variant was superior, but that image-only classification showed restricted discriminative performance in this clinical population.

The study has several limitations. It was retrospective and single center, and external validation was not performed. The reference label was based on routine clinical testing rather than a harmonized research protocol. In particular, serum anti-*H. pylori* IgG and the ^13^C urea breath test do not provide identical biological information: serology may remain positive after previous exposure or eradication, whereas the urea breath test more directly reflects current urease activity. This heterogeneity may have introduced label noise and may partly explain the limited image-only discrimination observed in this study.

The dataset size was modest for deep learning, reflecting the practical difficulty of assembling a clinically specific cohort of gastric cancer patients with available *H. pylori* testing and analyzable endoscopic images. Although patient-level splitting reduced the risk of data leakage, the limited number of patients and images increases the risk of overfitting and imprecise performance estimates. In addition, the number of images per patient was limited, and no external cohort was available to assess generalizability. Detailed subgroup analyses according to cancer stage, tumor location, histology, atrophy, intestinal metaplasia, or eradication history were not performed because the study was designed as a brief image-only report and because further stratification would have produced small and unstable subgroups. Because the models showed limited discrimination, we did not interpret model attention patterns or perform formal explainability analysis as evidence of specific mucosal features. Future work in this project will therefore focus on prospective collection of a new gastric cancer cohort with standardized image acquisition, harmonized *H. pylori* testing, and predefined clinical annotations, including tumor characteristics, mucosal background, and eradication history, followed by temporal or external validation.

Future work may benefit from several refinements. First, *H. pylori* status may need to be modeled in more than two states, for example, never infected, currently infected, and past infection (15). Second, more uniform reference standards would reduce label ambiguity. Third, video frames or broader endoscopic sampling might capture a more representative mucosal phenotype than a small number of still images, although this would also require careful patient-level separation to avoid leakage. Finally, multimodal approaches that integrate endoscopic images with eradication history, pathology, or other clinical variables may be more appropriate than image-only classification in gastric cancer patients.

In summary, this study does not suggest that endoscopic AI for *H. pylori* is without value. Rather, it shows that performance may degrade substantially in gastric cancer patients, where visual confounding and label ambiguity are both high. This limitation is itself informative and should help define more realistic expectations for future diagnostic and screening-oriented AI research in upper gastrointestinal endoscopy.

## Data Availability

The raw data supporting the conclusions of this article will be made available by the authors, without undue reservation.
